# Differential Transcriptomic Response in the Spleen and Head Kidney Following Vaccination and Infection of Asian Seabass with *Streptococcus iniae*

**DOI:** 10.1371/journal.pone.0099128

**Published:** 2014-07-03

**Authors:** Junhui Jiang, Masato Miyata, Candy Chan, Si Yan Ngoh, Woei Chang Liew, Jolly M. Saju, Kah Sing Ng, Fong Sian Wong, Yeng Sheng Lee, Siow Foong Chang, László Orbán

**Affiliations:** 1 Reproductive Genomics Group, Strategic Research Program, Temasek Life Sciences Laboratory, National University of Singapore, Singapore, Republic of Singapore; 2 Agri-Food and Veterinary Authority of Singapore, Singapore, Republic of Singapore; 3 Department of Biological Sciences, National University of Singapore, Singapore, Republic of Singapore; 4 MSD Animal Health Innovation, Singapore, Republic of Singapore; 5 School of Biological Sciences, Nanyang Technological University, Singapore, Republic of Singapore; 6 Department of Animal Sciences and Animal Husbandry, Georgikon Faculty, University of Pannonia, Keszthely, Hungary; 7 Centre for Comparative Genomics, Murdoch University, Murdoch, Australia; Louisiana State University School of Veterinary, United States of America

## Abstract

Vaccination is an important strategy in the protection of aquaculture species from major diseases. However, we still do not have a good understanding of the mechanisms underlying vaccine-induced disease resistance. This is further complicated by the presence of several lymphoid organs that play different roles when mounting an immune response. In this study, we attempt to elucidate some of these mechanisms using a microarray-based approach. Asian seabass (*Lates calcarifer*) were vaccinated against *Streptococcus iniae* and the transcriptomic changes within the spleen and head kidney at one and seven days post-vaccination were profiled. We subsequently challenged the seabass at three weeks post-vaccination with live *S. iniae* and similarly profiled the transcriptomes of the two organs after the challenge. We found that vaccination induced an early, but transient transcriptomic change in the spleens and a delayed response in the head kidneys, which became more similar to one another compared to un-vaccinated ones. When challenged with the pathogen, the spleen, but not the head kidneys, responded transcriptomically at 25–29 hours post-challenge. A unique set of genes, in particular those involved in the activation of NF-κB signaling, was up-regulated in the vaccinated spleens upon pathogen challenge but not in the un-vaccinated spleens. A semi-quantitative PCR detection of *S. iniae* using metagenomic DNA extracted from the water containing the seabass also revealed that vaccination resulted in reduction of pathogen shedding. This result indicated that vaccination not only led to a successful immune defense against the infection, but also reduced the chances for horizontal transmission of the pathogen. In conclusion, we have provided a transcriptomic analysis of how the teleost spleen and head kidneys responded to vaccination and subsequent infection. The different responses from the two organs are suggestive of their unique roles in establishing a vaccine-induced disease resistance.

## Introduction

Asian seabass (*Lates calcarifer*), also commonly known as barramundi, is a popular aquaculture species in Southeast Asia and Australia. However, disease outbreaks pose a major constraint to the development of Asian seabass aquaculture [1]–[5].

One of the major diseases affecting Asian seabass and other cultured marine teleosts is streptococcosis that has wide geographic occurrences and is not limited by water salinities [6]. This disease can result in both chronic low-grade mortalities as well as acute outbreaks resulting in overnight mass mortalities [7], [8]. The most frequent causative pathogen of streptococcosis in Asian seabass is *Streptococcus iniae* [9]. This disease is also a region-wide problem in Asian seabass culture here in Southeast Asia [10], [11]. Fortunately, several types of vaccines, including DNA-based, live attenuated and formalin-killed ones, have been developed against this disease [12]–[14]. While vaccination has become an important strategy in the management of this disease, we still do not have a good understanding of the mechanisms underlying the vaccine-induced disease resistance.

With the advent of RNA-seq technology and greater availability of expression microarrays, there are more studies into the transcriptomic response of host immune organs to vaccinations and/or disease infections on humans, model organisms and livestock [15]–[21]. Similar studies have also been carried out for aquaculture species using both traditional real-time RT-PCR methods or with custom expression microarrays [22]–[31]. It is hoped that through transcriptomic profiling, pathways or genes associated with protective immune response elicited by vaccines and host resistance can be identified with the eventual goals of generating biomarkers that can predict immunization success and improve vaccine immunogenicity [32], [33].

Hence, in this study we attempt to elucidate some of the pathways involved in the immune response to vaccination and subsequent disease challenge using a transcriptomic approach. At the same time, we are trying to learn more about the roles of the teleost spleen and head kidney in immunity. Both the spleen and head kidney are well-known lymphoid organs of the Asian seabass and are found to be developed at 2 days post hatching and are often rich in melano-macrophage centers, reflecting their immune-related roles [34].

In this study, we vaccinated, via peritoneal injection, three months-old Asian seabass using a commercially available *S. iniae* vaccine. Spleen and head kidney samples were collected at one and seven days post vaccination for transcriptomic analysis. Subsequently, a pathogen challenge was carried out three weeks later and spleen and head kidneys were sampled at 25–29 hours post challenge for transcriptomic analysis again. We showed that the spleen responded early, but transiently, to the vaccination and several genes involved in cell proliferation were found to be up-regulated. On the other hand, there was a delayed response of the head kidney to the vaccination that resulted in the increased homogeneity of their transcriptomes. In the subsequent acute disease infection, we found that only the spleen responded with changes in gene expressions that corroborated the activation of T cell-mediated adaptive immunity. In addition, a semi-quantitative PCR detection of *Streptococcus iniae* using metagenomic DNA extracted directly from the holding water of the challenged seabass showed that vaccination resulted in reduction of pathogen shedding.

## Materials and Methods

### 2.1. Ethics Statement

This study and all procedures were approved by Temasek Life Sciences Laboratory Institutional Animal Care and Use Committee (approval ID: TLL(F)-10-003) for experiments carried out at Temasek Life Sciences Laboratory (License for Animal Research Facility No. VR016) and MSD Animal Health Innovation Pte Ltd Institutional Animal Care and Use Committee (approval ID: Project EXT-EXP 05 Aug 2011) for experiments carried out at MSD Animal Health Innovation Pte Ltd (License for Animal Research Facility No. VR001). All animal handling protocols comply with guidelines set by the National Advisory Committee on Laboratory Animal Research (NACLAR) for the care and use of animals for scientific purposes in Singapore. The Asian seabass were housed in appropriate containers with aeration during the experiment and were sacrificed or euthanized by overdose of Tricaine methane-sulfonate (MS-222; at least 300 mg/L for over 10 minutes until total loss of gill movement) following AVMA (American Veterinary Medical Association) guideline for euthanasia of animals.

### 2.2. Animals, experimental design and sampling

Three-month-old juvenile Asian seabass (*Lates calcarifer*) weighing about 30 g were sourced from the Marine Aquaculture Centre of the Agri-food and Veterinary Authority of Singapore and housed in aerated rectangular tanks under 28–32°C and 12L:12D cycle conditions.

**Figure 1:**
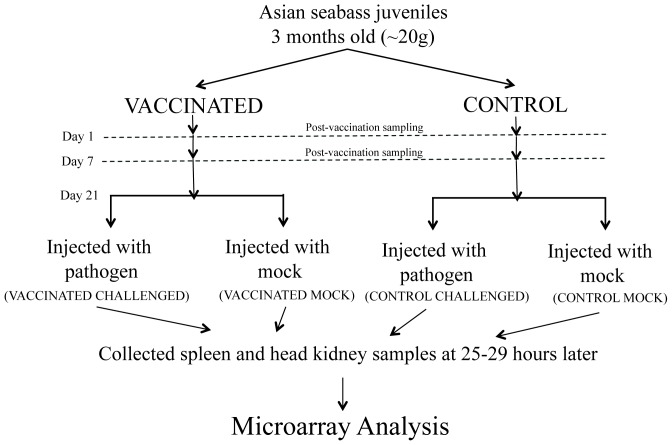
Flow chart of experimental setup. Spleen and head kidney samples were collected from a total of three time points (Day 1 and Day 7 post-vaccination; and 25–29 hours post-challenge).

A total of 210 individuals were used in this experiment. The experimental setup is summarized in **Fig. 1**. One group of juvenile seabass (105 individuals) was vaccinated against *Streptococcus iniae* via peritoneal injection (from here on termed ‘**vaccinated**’) with Norvax Strep Si (MSD Animal Health) at a dose of 0.1 ml/fish, while another group (105 individuals) was mock-vaccinated with equivalent volume of 0.01 M PBS with 1.5% saline (from here on termed ‘**control**’). To minimize pain during the injection, the fish were sedated with 30 parts per million (ppm) AQUI-S (50% isoeugenol; Aqui-S New Zealand Ltd). Subsequently, at Day 1 and Day 7 post-vaccination, 15 individuals were randomly sacrificed at each time point and from each group for spleen and head kidney collection. After the vaccination, the fish were housed in aerated rectangular tanks under 26–30°C and 14L:10D cycle conditions.

At 21 days post-vaccination, *S. iniae* challenge was carried out. From each of the vaccinated and control groups, 48 and 50 individuals, respectively, were challenged with live pathogen via peritoneal injection (*S. iniae* suspended in bacterial culture broth diluted to 4.6×10^7^ colony forming unit per milliliter (cfu/ml), 0.1 ml/fish), while 25 individuals from each group were mock-challenged using an equivalent volume of 0.01 M PBS with 1.5% saline. Similarly, to minimize pain, the fish were sedated with AQUI-S during the injection. The four groups of seabass (from here on termed ‘**vaccinated challenged**’, ‘**vaccinated mock**’, ‘**control challenged**’ and ‘**control mock**’) were kept as four separate groups. They were monitored hourly during the day from 8 AM until 10 PM. Individuals found displaying abnormal swimming behavior were considered moribund or dead for the purpose of the survival study, recorded and humanely euthanized. Any specimen found dead the next morning was recorded and immediately removed. Survival analysis was carried out using the statistical software GraphPad Prism 6.

Twelve individuals from each of the four groups (total: 48 seabass) were sacrificed at 9–13 hours post challenge (hpc) and another twelve from each group were sacrificed at 25–29 hpc for spleen and head kidney collection. The experiment was terminated at 48 hpc (after the second morning) and any surviving seabass were euthanized.

### 2.3. Semi-quantitative PCR detection of bacteria from water samples

Water samples were collected in autoclaved glass bottles in 100 ml quantities. The water was vacuum-filtered through a 0.22 µm polyethersulfone filter and stored at −20°C prior to DNA extraction. Metagenomic DNA was extracted from the filter using the MoBio PowerWater DNA Isolation Kit (MoBio Laboratories). The brains of *S. iniae*-infected seabass were collected to serve as positive controls and the genomic DNA was extracted using the phenol-chloroform method. Genomic DNA from competent *E. coli* cells was extracted using Genomic DNA Mini Kit (Geneaid Biotech Ltd) and used as negative controls.

In order to detect the presence of *S. iniae*, a specific nested PCR protocol was carried out according to Berridge *et al.* (1998) [35]. First, a non-specific 16S-23S PCR reaction with forward primer (A1 -5′AGTCGTAACAAGGTAAGCCG3′) and reverse primer (B1 - 5′C T/C A/G T/C TGCCAAGCATCCACT3′) were cycled 35 times at 94°C for 1 min, 50°C for 1 min, and 72°C for 1 min, with a final extension for 5 min at 72°C [35]. Subsequently, the PCR product from the above reaction was used as template for a round of *S. iniae* specific PCR reaction with forward primer (5′144 - 5′GGAAAGAGACGCAGTGTCAAAACAC3′) and reverse primer (3′516 – 5′CTTACCTTAGCCCCAGTCTAAGGAC3′). The PCR cycling parameters for this reaction was similar to the previous one with modification of the annealing temperature from 50°C to 60°C. All PCR amplification products were examined by electrophoresis on a 1.5% agarose gel containing ethidium bromide. The expected product size for *S. iniae* specific reaction was 373 bp.

### 2.4. RNA extraction

Total RNA was extracted from spleen and head kidney tissues using the RNeasy Mini Kit (Qiagen) following the manufacturer's instructions. RNA quantity and quality was determined using spectrophotometer (NanoDrop) and Agilent 2100 Bioanalyzer (Agilent Technologies) respectively. Only RNA samples with RIN number >8 were used for microarray analysis.

### 2.5. Microarray hybridization and analysis

Microarray analyses were carried out on Agilent SurePrint G3 custom gene expression 8×60K oligonucleotide arrays (Cat No. G4102A). Eight microarray slides were used in this study and the data had been deposited into NCBI Gene Expression Omnibus database (**GSE51839**).

Total RNA (100 ng) was reverse transcribed and labeled using Agilent Low Input Quick Amp One Color labeling kit (Cat No. 5190-2305) according to the manufacturer's instructions. cRNA samples were labeled with cyanine 3-CTP and the amount of cyanine 3-labeled cRNA was determined using NanoDrop. Labeled cRNA samples with cRNA yields >0.825 µg and specific activity >6 pmol Cy3 per µg cRNA were used for hybridization.

Equal amounts of labeled target sample cRNA (600 ng) samples were hybridized to each individual array. Microarray hybridization conditions and washing procedures were performed as described in Agilent Gene Analysis protocols (One Color Microarray-Based Gene Expression Analysis, Version 6.5). Microarray slides were scanned using Profile AgilentG3_GX_1Color and the scan data were extracted using Agilent Feature Extraction Software.

Partek Genomic Suite (v6.6) was used to analyze the data. Microarray data were quantile normalized and log_2_ transformed for statistical analysis. Genes of interest that showed differential expression (>1.5-fold; p-value<0.05) were annotated further using KEGG Automatic Annotation Server (KEGG KAAS) through the single-directional best hit method [36].

### 2.6. Real-time RT-PCR

Real-time RT-PCR was carried out using the BioMark HD system (Fluidigm Corporation). Total RNA from spleen and head kidney (800 ng) were reverse transcribed using iScript cDNA Synthesis Kit (Bio-Rad Laboratories) following the manufacturer's instructions. Specific target amplification was carried out on the cDNA using TaqMan PreAmp Master Mix (Applied Biosystems PN 4361128) and the products were loaded onto the Fluidigm's Dynamic Array Integrated Fluidic Circuits (IFC) according to Fluidigm's EvaGreen DNA Binding Dye protocols. Quadruplicates were analyzed for each biological sample. The sequences of the primers used are listed in **Table S1**.

### 2.7. Gram staining of spleen and head kidney sections

Gram staining was carried out on cryosections of spleens and head kidneys. The samples were fixed overnight in 4% paraformaldehyde and followed by soaking in 30% sucrose for 6 hours at 4°C. The samples were then frozen in Jung tissue freezing and sectioned at 16 µm thickness by cryostat (Leica). Gram staining was carried by applying crystal violet staining reagent (2% weight per volume (w/v) crystal violet and 0.8% w/v ammonium oxalate in 20% ethanol) to the sections for 1 min and then washed in tap water briefly. This was followed by staining with Gram's Iodine (33% w/v iodine and 66% w/v potassium iodide) for 2 min and brief washing in tap water before decolourization in 95% ethanol and counterstaining using safranin (0.25% w/v safranin O in 10% ethanol).

## Results

### 3.1. Vaccination reduced mortalities and pathogen shedding in a pathogen challenge test

**Figure 2:**
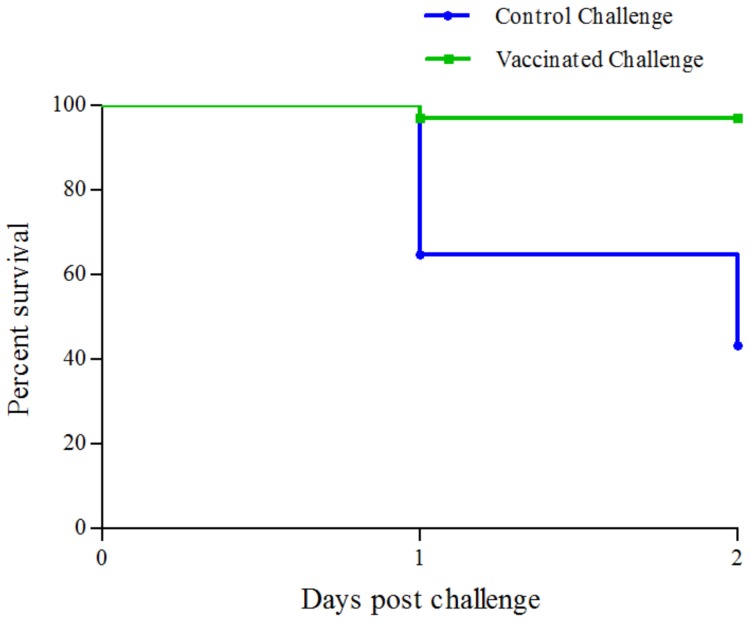
Survival curves for control challenged and vaccinated challenged seabass. The vaccinated challenged seabass showed better survival than the control seabass as the survival curves were significantly different (log-rank (Mantel-Cox) test; *P*<10^−4^).

For both the ‘vaccinated challenged’ and ‘control challenged’ groups, with starting number of 48 and 50 individuals, respectively, 24 live seabass were sacrificed from each group for sample collection – 12 from each group at 9–13 hours post-challenge (hpc; not used in this study) and 12 from each group at 25–29 hpc. From the rest, only a single seabass died in the ‘vaccinated challenged’ group, while 17 were lost from the ‘control challenged’ group by the time the experiment was terminated at 2 days post challenge. Survival analysis using the log-rank (Mantel-Cox) test indicated that the survival curve for the ‘control challenged’ group was significantly different from that of the ‘vaccinated challenged’ group (p-value <10^−4^) (**Fig. 2**). This showed that the three week period between the vaccination and challenge were sufficiently long for the adaptive immunity to develop in the seabass against *S. iniae*. In addition, the control seabass started to die at 24 hpc, indicating that critical immune responses had to be activated before then.

**Figure 3:**
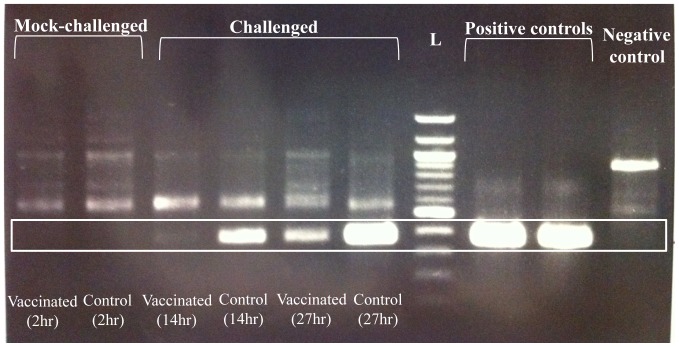
Semi-quantitative PCR detected variable amount of *Streptococcus iniae* from the water samples holding pathogen-challenged fish. The boxed region indicates the expected band size from *S. iniae* specific reaction (373 bp). No *S. iniae* band could be detected from the two ‘mock challenged’ groups and the negative control. The ‘control challenged’ group at 27 hours post-challenge (hpc) had the most intense band followed by ‘control challenged’ group at 14 hpc, ‘vaccinated challenged’ group at 27 hpc and ‘vaccinated challenged’ group at 14 hpc. The positive controls were from total genomic DNA extracted from brain of infected seabass. The negative controls were *E.coli* (XL-1 Blue) genomic DNA. L indicates 100 bp DNA ladder (NEB). Brackets indicate hours post challenge (hpc).

We also carried out a semi-quantitative PCR detection of *S. iniae* from each of the water samples in which the four groups of seabass (i.e. ‘vaccinated challenged’, ‘vaccinated mock’, ‘control challenged’ and ‘control mock’) were kept (**Fig. 3**). A faint band of the expected product size for *S. iniae*-specific reaction could be detected from the water of ‘vaccinated challenged’ group at 14 hours post-challenge, while a much brighter band was observed from the ‘control challenged’ group at the same time point. At a later time point (37 hpc), the intensity of the band amplified from the water increased for both groups with the ‘control challenge’ group having a stronger band than that of the ‘vaccinated challenged’ group, which was still less intense than the ‘control challenged’ group at 14 hpc. This result indicated that *S. iniae* was present in the water holding both the ‘vaccinated challenged’ and ‘control challenged’ groups and the amount of *S. iniae* increased over time for both groups. However, the amount of *S. iniae* in the ‘vaccinated challenged’ group still remained less than that of the ‘control challenged’ group.

### 3.2. The presence of bacteria was confirmed in spleen and head kidney of control (un-vaccinated) seabass after pathogen challenge

**Figure 4:**
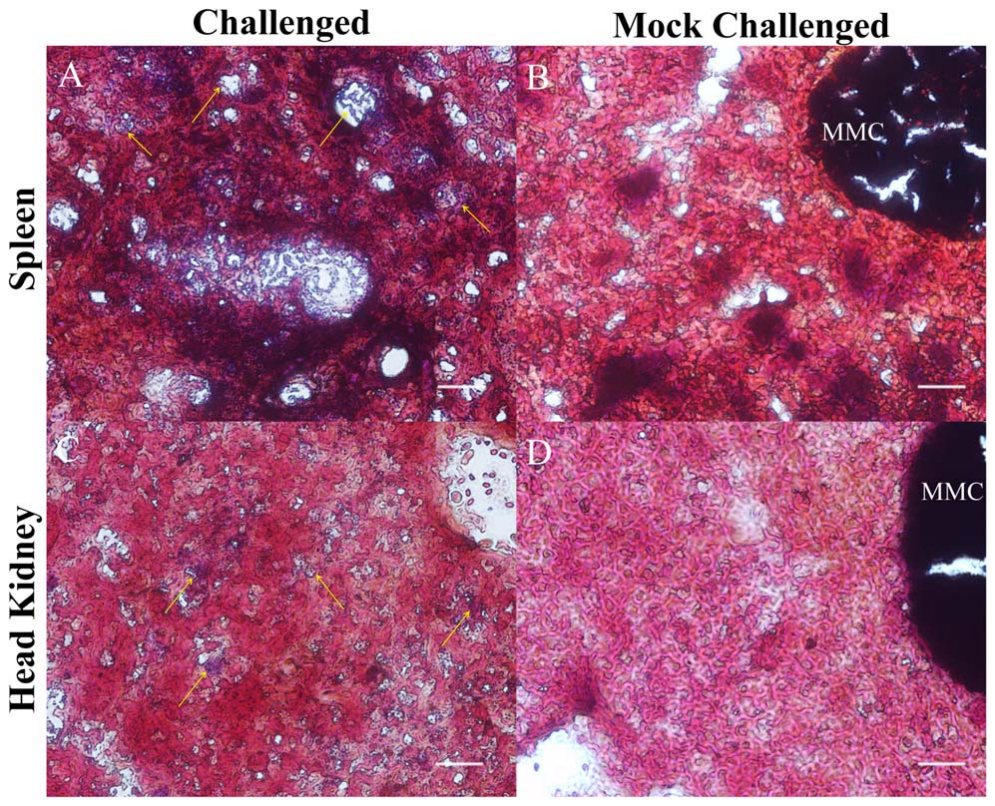
Gram staining of spleen and head kidney sections. Chain-like coccoid Gram-positive bacteria were present in the spleens (A) and head kidneys (C) of the ‘control challenged’ seabass, but not in the spleens (B) and head kidneys (D) of ‘control mock’ seabass. Arrows indicate bacteria. MMC - melano-macrophage center. Scale bar −20 µm.

Gram staining of spleens and head kidneys sections from control challenged seabass revealed the presence of gram-positive bacteria in these organs (**Fig. 4**). This confirmed that even though the fish were infected artificially, the bacteria remained viable and were able to spread into the spleens and head kidneys by 25–29 hours post challenge. For the vaccinated challenged groups, we could not find chain-like coccoid bacteria representative of *Streptococcus* within the spleens or head kidney samples, but could not rule out the presence of bacteria in the ‘vaccinated challenged’ spleens and head kidneys.

### 3.3. Vaccination induced only transient change in the spleen transcriptome that disappeared by Day 7

**Figure 5:**
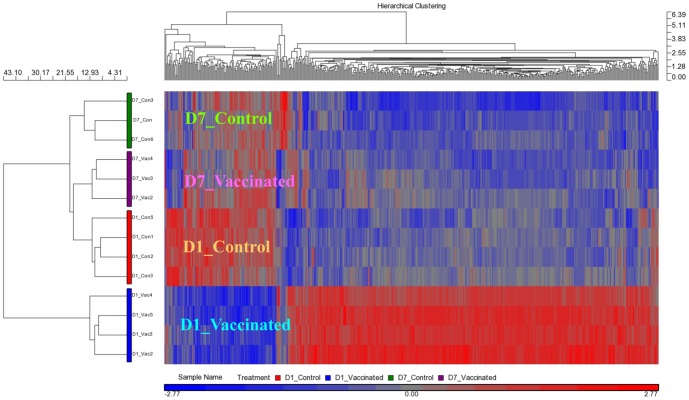
Vaccination induced a transient change in spleen transcriptome. The Day 1 post-vaccination spleens (D1_Vaccinated) clustered in their own primary clade while the Day 7 post-vaccination spleens (D7_Vaccinated) and the control spleens at Day 1 and Day 7 (D1_Control and D7_Control, respectively) clustered together in another primary clade. The D7_Vaccinated, D1_Control and D7_Control spleens were clustered separately in their own secondary clades, highlighting their distinct transcriptome profiles even though the expression of individual genes may not have shown significant change between the three groups. The hierarchical clustering map was generated using 498 probes that showed p-value with false discovery rate (FDR) (treatment – vaccination status and days post-vaccination) <0.05 in a one-way Anova analysis. The color bar at the bottom indicates the relative expression level of transcripts. Red boxes indicate high expression, whereas the blue ones label low expression.

At Day 1 post-vaccination, there were 938 differentially expressed transcripts (DETs) between spleens from the ‘vaccinated’ and ‘control’ groups. However, at Day 7 post-vaccination, this was reduced to only a single DET (**Table 1**). No transcript showed significant difference between Day 1 and Day 7 control spleens, while over 300 DETs were found between Day 1 and Day 7 vaccinated spleens. The difference between Day 1 and Day 7 vaccinated spleens was also reflected in the hierarchical clustering map (**Fig. 5**).

**Table 1:** The number of Differentially Expressed Transcripts* between vaccinated and control spleens at Day 1 (D1) and Day 7 (D7) post-vaccination.

Comparison	Upregulated	Downregulated	Total
D1 Vaccinated vs D1 Control	485	453	938
D7 Vaccinated vs D7 Control	1	0	1
D7 Vacinated vs D1 Vaccinated	51	280	331
D7 Control vs D1 Control	0	0	0

*p-value FDR<0.05; fold-change ≥1.5 or ≤−1.5.

Under the hierarchical clustering map, Day 1 vaccinated spleens clustered separately from the other three groups of spleens. Notably, Day 7 vaccinated spleens were clustered together with the control spleens, indicating that their transcriptomes were more similar to the control spleens than those of the Day 1 vaccinated spleens (**Fig 5**). Taken together, the results showed that the change in the transcriptome profile of the spleen brought about by the vaccination was transient.

A KEGG analysis of the 485 DETs that were up-regulated in Day 1 vaccinated spleens compared to Day 1 control spleens indicated that many of these transcripts represented genes that were associated with pathways involved in DNA replication, cell cycle, Fanconi anemia pathway and purine and pyrimidine metabolism. Genes involved in proteolysis, phagocytosis and apoptosis were also up-regulated (**Table 2**; see **Table S2** for the complete list of 938 DETs and **Table S3** for the complete list of genes involved in the above-mentioned pathways). Only seven immune-related genes were found to be up-regulated as a result of the vaccination and two such examples were - *complement component 7* (16.9 fold-change; p-value <0.001) and *interleukin 12b* (4.0 fold-change; p-value  = 0.006) (**Table S2**).

**Table 2:** The list of KEGG pathways with member genes up-regulated* in the spleen at one day post-vaccination.

KEGG	KEGG Pathway	No. of genes
ko03050	Proteasome	11
ko04145	Phagosome	5
ko04115	p53 signaling	10
ko03460	Fanconi anemia pathway	12
ko04110	Cell cycle	31
ko03030	DNA replication	25
ko00230	Purine metabolism	14
ko00240	Pyrimidine metabolism	17

*p-value FDR<0.05; fold-change ≥1.5.

### 3.4. Upon pathogen challenge, spleens from vaccinated seabass showed an up-regulation of a specific set of immune response genes not found in controls

**Figure 6:**
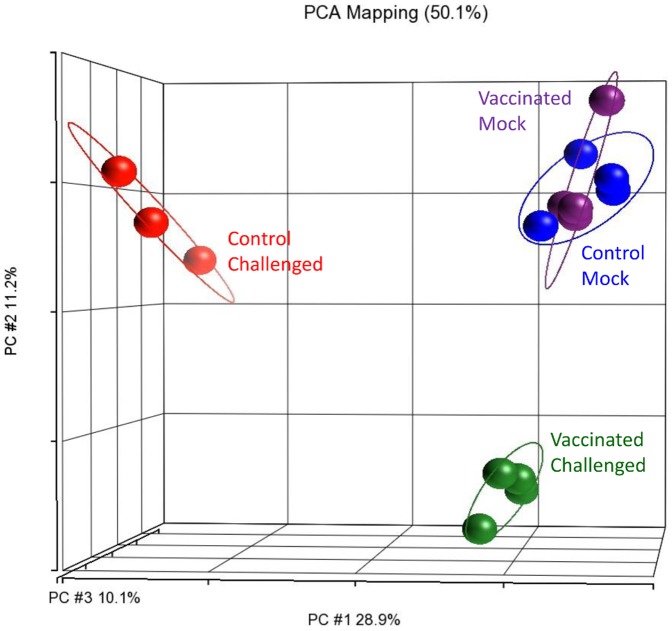
‘Mock challenged’ spleens clustered together regardless of vaccination background under a principal component analysis plot. The ‘mock challenged’ spleens exhibited similar transcriptome profiles at 22 days post-vaccination, while the ‘control challenged’ and ‘vaccinated challenged’ ones grouped separately, reflecting their transcriptomic distinctness from one another and from the ‘mock challenged’ spleens. Each sphere represents the transcriptomic profile of the spleen of one seabass individual.

The pathogen challenge took place three weeks after the vaccination and spleen samples were collected at 25–29 hpc. A principal component analysis of the spleen transcriptomes showed that the ‘mock challenged’ spleens, whether vaccinated or control, grouped together (**Fig. 6**). This indicated that their transcriptomes were similar. In addition, there was also no DET found between these two mock-challenged groups (**Table 3**). This result further confirmed the transient effect of vaccination on the spleen transcriptome.

**Table 3:** The number of Differentially Expressed Transcripts* between the four groups of spleens at 25–29 hours post challenge.

Comparison	Upregulated	Downregulated	Total
Vaccinated Challenged vs Vaccinated Mock	438**	538	976
Control Challenged vs Control Mock	5,285	6,872	12,157
Vaccinated Mock vs Control Mock	0	0	0

*(p-value FDR<0.05; fold-change ≥1.5 or ≤−1.5).

**See **Tables S4 and S5** for the gene lists.

**Figure 7:**
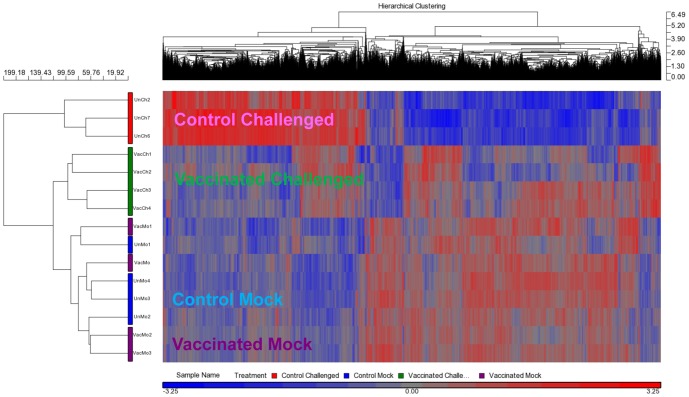
‘Control challenged’ spleen had greater change in transcriptome profile compared to ‘vaccinated challenged’ spleen. The transcriptomic profiles of the ‘vaccinated challenged’ spleens at 22 days post-vaccination were more similar to the ‘control mock’ and ‘vaccinated mock’ spleens. The ‘vaccinated challenged’ spleens clustered together with the ‘mock challenged’ ones in the same primary clade, but in their own sub-clade, while the ‘control challenged’ seabass clustered into another major clade. The hierarchical clustering map was generated using 10,331 probes that showed a p-value with FDR (treatment) <0.05 in a one-way Anova analysis.

On the other hand, the ‘vaccinated challenged’ and ‘control challenged’ spleens clustered away from one another and also away from the two ‘mock challenged’ groups (**Fig. 6**). Following pathogen challenge, spleens from ‘control challenged’ seabass showed a drastic change in their transcriptome profile with over 12,000 DETs when compared to the ‘control mock’ (**Table 3**). In the ‘vaccinated challenged’ seabass, the change was less drastic and there were only 976 DETs when compared to the ‘vaccinated mock’ (**Table 3**). In addition, under the hierarchical clustering map, the vaccinated challenged spleens clustered together with the mock challenged samples in the same primary clade while the control challenged seabass clustered into another primary clade (**Fig. 7**).

**Figure 8:**
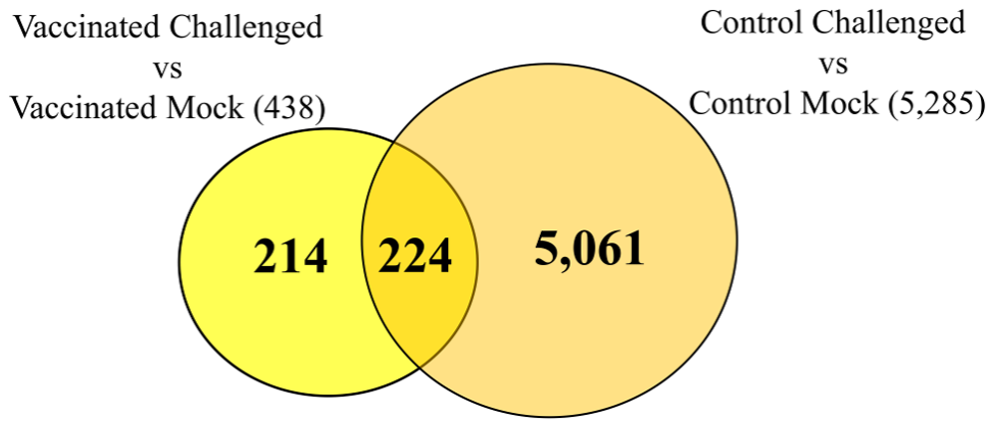
Up-regulated transcripts after pathogen challenge in the spleen. The Venn diagram shows the number of up-regulated transcripts when comparing the ‘challenged’ spleens against ‘mock challenged’ ones under the vaccinated and control (un-vaccinated) backgrounds. A total of 214 transcripts were uniquely up-regulated in the vaccinated background, while not found among the 5,285 that were up-regulated upon challenge in the un-vaccinated background. (For the list of these 214 transcripts see Supplementary Table S4).

There were 214 DETs that showed up-regulation upon pathogen challenge in the vaccinated spleens, but not in the controls when compared against their respective ‘mock challenged’ spleens (**Fig. 8**). Among these 214 DETs, nine were genes involved in immune-related pathways and eight of them were further validated by real-time RT-PCR. Data from the validation test also confirmed that these eight genes do not show any up-regulation in the ‘control challenged’ spleens indicating the possible use of these genes as biomarkers for response to *S. iniae* infection in a vaccinated seabass (**Table 4A**).

**Table 4:** List of genes tested by real-time RT-PCR for potential biomarkers of vaccination-induced response in spleen.

			Vaccinated Challenged vs Vaccinated Mock (Spleen)					Control Challenged vs Control Mock (Spleen)				
			Microarray		qRT-PCR		Validation*/Direction	Microarray		qRT-PCR		Validation/Direction
S/N	KO	Gene Symbol & Name	p-value	Fold-Change	p-value	Fold-Change		p-value	Fold-Change	p-value	Fold-Change	
**(A) Genes that are uniquely up-regulated in vaccinated challenged spleens**												
1	K05856	*lck; lymphocyte cell-specific protein tyrosine kinase*	0.039	1.96	0.003	2.68	Yes/Up	0.432	−1.18	0.278	1.51	Yes/NS**
2	K07360	*zap70; zeta-chain (TCR) associated protein kinase*	0.017	2.76	0.001	4.45	Yes/Up	0.039	−1.67	0.796	1.04	No
3	K10785	*trvb; T-cell receptor beta chain variable region*	0.019	2.29	7.9E-05	2.78	Yes/Up	0.221	−1.27	0.529	1.12	Yes/NS
4	K04179	*ccr4; C-C chemokine receptor type 4*	0.027	3.20	0.018	2.26	Yes/Up	0.125	−1.63	0.003	−3.47	No
5	K04182	*ccr7; C-C chemokine receptor type 7*	0.037	3.84	0.011	4.27	Yes/Up	0.014	−3.31	0.029	−2.59	Yes/Down
6	K04190	*cxcr5; C-X-C chemokine receptor type 5*	0.038	3.69	1.6E-04	3.91	Yes/Up	0.881	1.07	0.204	−1.45	Yes/NS
7	K07990	*sh2d1a; SH2 domain containing 1a*	0.031	3.37	0.007	3.10	Yes/Up	0.131	1.70	0.528	1.16	Yes/NS
8	K05398	*tlr1; toll-like receptor 1*	0.035	5.74	4.0E-04	6.40	Yes/Up	0.545	1.37	0.404	1.23	Yes/NS
9	K02161	*bcl2; B-cell leukemia/lymphoma 2*	0.049	4.04	0.177	10.12	No	0.211	−1.79	0.140	188.23	Yes/NS
**(B) Genes that are uniquely differentially expressed in control challenged spleens**												
1	K05496	*bmp3/3b; bone morphogenetic protein 3/3b*	0.886	−1.25	0.663	1.51	Yes/NS	0.001	−63.28	0.053	−3.33	No
2	NIL	*gas7l; growth arrest-specific 7-like* (By Blast)	0.242	4.12	0.330	1.65	Yes/NS	0.044	−6.57	0.049	−3.61	Yes/Down
3	K03173	*traf2; Tnf receptor-associated factor 2*	0.109	1.96	0.015	1.79	No	0.001	6.42	0.008	2.72	Yes/Up
4	K08617	*adamts1; ADAM metallopeptidase with thrombospondin type 1 motif, 1*	0.896	−1.56	0.105	10.04	Yes/NS	0.047	17.50	0.005	18.05	Yes/Up
5	K05148	*tnfrsf11b; tumor necrosis factor receptor superfamily member 11b*	0.632	1.94	0.083	6.83	Yes/NS	0.003	26.16	0.416	1.68	No
6	K10030	*il8; interleukin 8*	0.308	1.66	0.036	7.87	No	1.7E-04	35.09	0.001	13.82	Yes/Up
7	K05443	*il10; interleukin 10*	0.546	1.99	0.079	8.04	Yes/NS	0.001	42.96	0.281	5.86	No
8	K03996	*c7; complement component 7*	0.289	3.19	0.095	31.67	Yes/NS	0.001	54.71	3.5E-05	83.16	Yes/Up
9	K04387	*il1r2; interleukin 1 receptor type 2*	0.736	1.43	0.222	11.35	Yes/NS	4.1E-04	68.86	4.7E-06	46.69	Yes/Up
10	K04519	*il1b; interleukin 1b*	0.854	1.38	0.037	69.17	No	4.4E-04	300.64	8.4E-06	258.16	Yes/Up
11	K04358	*fgf; fibroblast growth factor*	0.988	1.10	0.113	15.21	Yes/NS	0.002	394.46	0.003	98.10	Yes/Up
12	K05625	*tgm2; transglutaminase 2*	0.542	1.76	0.184	6.83	Yes/NS	1.4E-04	472.68	0.014	383.82	Yes/Up

*Conditions for validation of microarray data by real-time RT-PCR: For significant differential expression, data from both platforms must show p-value ≤0.05 and fold-change≥1.5 or ≤−1.5 in the same direction.

**NS indicate no significant change (p-value >0.05 and/or fold-change <1.5 or >−1.5).

On the other hand, there were 5,061 DETs that showed up-regulation in the control spleens, but not in the vaccinated spleens, when compared against their respective ‘mock challenged’ counterparts (**Fig. 8**). These DETs included genes involved in numerous signaling pathways and immune pathways (**Table S6**). We have also tested 12 genes that were found by microarray to be differentially expressed in the ‘control challenged’ spleen, but not in the ‘vaccinated challenged’ spleens when compared against their respective ‘mock challenged’ counterparts. For these 24 microarray results (12 genes each for the two comparisons – ‘vaccinated challenged’ vs. ‘vaccinated mock’ and ‘control challenged’ vs. ‘control mock’), 18 were validated by real-time RT-PCR and six genes were further confirmed to be differentially expressed only in the ‘control challenged’ spleens and not in the ‘vaccinated challenged’ ones (**Table 4B**). Likewise, these six genes could be used as biomarkers for response to *S. iniae* infection in un-vaccinated Asian seabass.

In addition, up-regulated genes that were common to both vaccinated and control seabass upon challenge were involved in DNA replication and cell cycle (**Table S4 and Table S5**). This result was similar to that observed in the spleen at Day 1 post-vaccination.

### 3.5. Vaccination did not induce significant change to the head kidney transcriptome at Day 1 and Day 7

**Figure 9:**
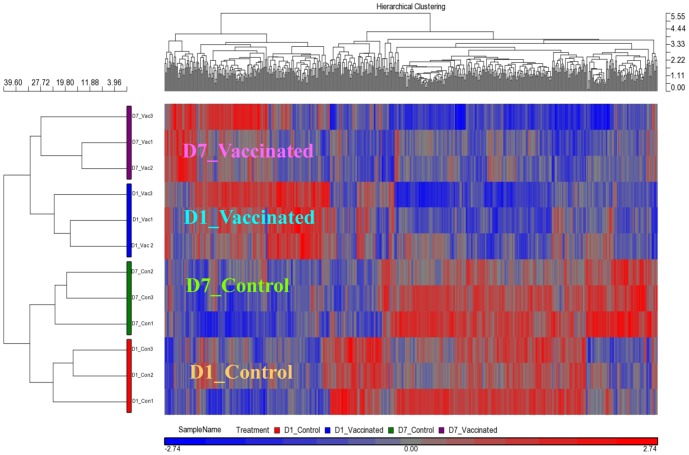
The head kidney samples were separated into two major clades according to the vaccination background. The head kidneys were then further separated based on the days post-vaccination within each of the two clades. The hierarchical clustering map was generated based on 620 genes with p-value (treatment) (unadjusted p-value) <0.01 in a one-way Anova analysis.

In the head kidney, there was no DET at Day 1 post vaccination while only three DETs were found at Day 7 when compared against the respective controls (**Table 5**). Although there were no or few DETs between the vaccinated and control head kidneys (p<0.05, fold change of at least 1.5-fold), a transcriptomic analysis using the hierarchical clustering map showed that the vaccinated and control head kidneys clustered separately into two primary clades (**Fig. 9**). This indicated that there were only subtle, but statistically insignificant differences in the overall transcriptomic profiles, when the expression levels of genes were compared between the vaccinated and control head kidneys.

**Table 5:** The number of Differentially Expressed Transcripts* between vaccinated and control head kidneys at Day 1 (D1) and Day 7 (D7) post vaccination.

Comparison	Upregulated	Downregulated	Total
D7 Vaccinated vs D1 Vaccinated	0	0	0
D1 vaccinated vs D1 Control	0	0	0
D7 Vaccinated vs D7 Control	1	2	3
Vaccinated vs Control	1	1	2

*p-value FDR<0.05; fold-change ≥1.5 or ≤−1.5.

### 3.6. Vaccination, not the pathogen challenge, is the key factor in affecting head kidney transcriptome at 25–29 hours post challenge

**Figure 10:**
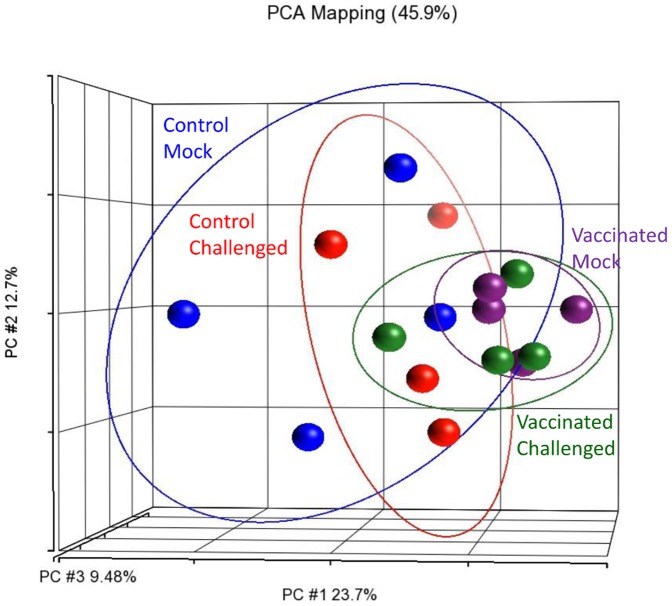
Vaccination is the key factor in affecting head kidney transcriptomes at 25–29 hours post challenge. A principal component analysis of head kidney transcriptomes showed that the transcriptome profiles of the controls were more dispersed than those of the vaccinated ones which also clustered together.

In a distinct contrast to the spleen, there was almost no DET between the ‘challenged’ and ‘mock challenged’ head kidneys (whether vaccinated or not; **Table 6**). However, when we compared vaccinated head kidneys against control ones (ignoring the challenged or mock challenged treatment), there were over 500 DETs (**Table S7**). There seemed to be no effect of the pathogen challenge in the head kidney at 25–29 hpc. In addition, under the PCA plot, the control head kidneys (whether challenged or mock challenged) were relatively dispersed compared to the vaccinated head kidneys (**Fig. 10**). This indicated that the transcriptome profiles of the control head kidneys were somewhat dissimilar to one another while those of the vaccinated head kidneys became more uniform.

**Table 6:** The number of Differentially Expressed Transcripts*s between the various groups of head kidneys at 25–29 hours post challenge.

Comparison	Upregulated	Downregulated	Total
Vaccinated vs Control	318	259	577
Vaccinated Challenged vs Vaccinated Mock	0	0	0
Control Challenged vs Control Mock	1	0	1

*p-value FDR<0.05; fold-change ≥1.5 or ≤−1.5.

Both at Day 1 and Day 7 post vaccination, there was minimal change between the head kidney transcriptomes of vaccinated and control groups. However, at three weeks post-vaccination, the number of DETs increased drastically, suggesting that the effects of vaccination on the head kidney took more than seven days to manifest.

## Discussion

In this study, transcriptome profiling of the spleen and head kidney of Asian seabass revealed a number of interesting aspects in the responses of these two lymphoid organs to vaccination and subsequent acute disease infection.

First, vaccination induced only a transient effect on gene expression in the spleen that disappeared by Day 7. A KEGG analysis of the genes that were up-regulated in the vaccinated spleens at Day 1 identified genes involved in cell proliferation, proteolysis, phagocytosis and apoptosis. On the other hand, the effect of vaccination on the head kidney took more than seven days to manifest as the change in transcriptome profile was detected only at three weeks post vaccination. Second, we found that vaccination resulted in the head kidneys having a more similar transcriptome profile to one another compared to those of controls.

Based on the results, we speculate that what we had observed in this study could possibly be a consequence in the differential compartmentalization of B cells, plasma cells and T cells between the spleen and head kidney. The early transient transcriptomic response of spleen towards vaccination and the delayed response of head kidneys suggest that the trapping of antigens (vaccine) and the subsequent differentiation and proliferation of naïve non-circulating B cells took place within the spleen. This would also explain why we saw mainly genes involved in cell proliferation, proteolysis, phagocytosis and apoptosis to be transiently up-regulated in the spleen. These memory B cells then subsequently migrate towards the head kidney niche where they further differentiate into long-lived plasma cells (antibody secreting cells). The relative similarity of the head kidney transcriptomes among vaccinated seabass compared to control seabass could hence be a consequence of the presence of a large mono-population of long-live plasma cells. This pattern of B cell differentiation, proliferation and migration was similarly observed in a study based on ELISPOT assays and carried out on rainbow trout (*Oncorhynchus mykiss*) subjected to an exposure of bacterial-based antigens [37]. Our result also implied that vaccinated seabass could still possibly be vulnerable to a bacterial infection shortly after vaccination (at least within one week) since long-lived plasma cells need some time to establish themselves in the head kidneys.

In the second part of our study, a disease challenge was carried out at three weeks post-vaccination. Our results showed that transcriptomic response could be observed in the spleen, but not in the head kidney, at 25–29 hours post-challenge despite the presence of bacteria in both organs. This indicates that the absence of transcriptomic response in the head kidney is not due to the failure of bacteria to reach the head kidney.

In addition, we found a specific set of genes that were up-regulated only in the spleens of vaccinated seabass, but not in those of control seabass at 25–29 hours post-challenge (**Table 4**). These genes were found to be involved in processes such as NF-κB signaling, chemokine signaling and toll like receptor signaling, and they have the potential to be used as biomarkers for the prediction of successful immune defense against acute *S. iniae* infection.

NF-κB signaling is a highly complex signal transduction system involved in numerous physiological functions including innate and adaptive immune responses through the transcription of target genes [38], [39]. Crucially, lymphocyte cell-specific protein tyrosine kinase (Lck) and zeta-chain (TCR) associated protein kinase (Zap70) are the key cytoplasmic protein kinases that interact sequentially with the T cell antigen receptor to activate classical NF-κB signaling through the phosphorylation of IκB (NF-κB inhibitor protein) [40]–[42]. The genes, *lck*, *zap70* and *trbv* (*T-cell receptor beta chain variable region*), were found to be up-regulated in the ‘vaccinated challenged’ spleens, but not in the ‘control challenged’ ones (**Table 4**). This indicates that the timely activation of NF-κB signaling, possibly in the T cells, may be critical in the survival of the seabass to the bacterial infection. Indeed NF-κB has been suggested to be a potential therapeutic target in microbial infections [43].

Similar to NF-κB signaling, chemokines and their receptors are also involved in multiple physiological roles including pro-inflammatory responses. While different chemokines may have overlapping functions or can bind to the same receptor, specific outcomes can also be generated through the interaction between receptor N-terminal domain and N-loop of chemokines and as well as through different types of receptor homo- or heterodimerization [44], [45]. Interestingly, different sets of chemokine receptor genes were found to be up-regulated in the ‘vaccinated challenged’ and ‘control challenged’ spleens. In the ‘vaccinated challenged’ spleens, *C-C chemokine receptor type 4* (*ccr4*), *C-C chemokine receptor type 7* (*ccr7*) and *C-X-C chemokine receptor type 5* (*cxcr5*) were up-regulated, whereas in the ‘control challenged’ spleens, a different set including *C-C chemokine receptor type 11* (*ccr11*), *C-X-C chemokine receptor type 3* (*cxcr3*) and *chemokine (C motif) receptor 1* (*xcr1*) was up-regulated (**Table 4** **, Table S5**). In particular, *ccr4* and *ccr7* were also found to be down-regulated in ‘control challenged’ spleens instead. Paradoxically, Ccr4 has been shown to play a detrimental role in bacterial infection as CCR4^−/−^ mice were resistant against lipopolysaccharide-induced lethality, infection by pathogenic *E.coli* and septic peritonitis induced by cecal ligation and puncture [46]–[48]. On the other hand, in mammalian models, Ccr7 is expressed in T cells and is required for the chemotaxis of such immune cells towards lymphoid organs such as spleen [49], [50]. Taken together, the up-regulation of *ccr7*, *lck*, *zap70* and *trbv* suggests that a rapid T cell-mediated adaptive immune response was activated in the vaccinated spleens upon the pathogen challenge. We hypothesize that T cell-mediated adaptive immune response was activated in the spleen shortly after the pathogen challenge and this contributed to a successful immune defense. The absence of transcriptomic response within the head kidney at 25–29 hours could also suggest that this T cell-mediated response does not occur there. For future studies, it would be informative to observe the transcriptomic profile of the head kidneys at 3 weeks post-challenge to find out whether we observe a similar result to the one seen after the initial vaccination. This would indicate that the head kidney possibly serves as a niche for the establishment of long-live plasma cells.

We have also detected the up-regulation of different toll-like receptors in the vaccinated and control spleens following the challenge. Toll-like receptors are involved in the innate immune responses through the recognition of conserved bacterial ligands/peptides and all toll-like receptor pathways also activate the downstream phosphorylation-dependent activation of NF-κB [51], [52]. Toll-like receptors have also been linked to the regulation of adaptive immune responses [53], [54]. *toll-like receptor 1* (*tlr1*), which was up-regulated by 6.4-fold in the ‘vaccinated challenged’ spleen, but not in the ‘control challenge’ one, forms a heterodimer with *toll-like receptor 2* (*tlr2*) for the recognition of specific bacterial ligands such as triacyl lipoproteins [55]. On the other hand, *toll-like receptor 5* (*tlr5*) was up-regulated by over 150-fold in the ‘control challenged’ spleens, but not in the ‘vaccinated challenged’ ones (**Table S5**). However, *tlr5* functions to identify flagellins of gram-negative bacteria, whereas *S. iniae* is gram positive and does not possess any flagella [56]. While this finding has been contrary to expectations, it was found that mucosal exposure to flagellins can protect mice from *S. pneumoniae* through Tlr5 signaling [57]. This is even though *S. pneumoniae* similarly do not possess any flagella. It was hypothesized by the authors that activation of Tlr5 signaling might possibly enhance alternate innate immune defenses against the pneumococcal infection. As such, we may be witnessing a similar phenomenon in un-vaccinated seabass against acute *Streptococcus* infection.

We have also found the up-regulation of *SH2 domain containing 1a* (*sh2d1a*) in ‘vaccinated challenged’ spleens, but not in ‘control challenged’ ones (**Table 4**). In humans, mutations of *SH2D1A* are the cause of the immunodeficiency disease, X-linked lymphoproliferative disease (XLP) and it has been found in mice that SH2D1A is required for the proper functioning of the primary and secondary immunoglobulin responses [58], [59]. It was also shown in mice that SH2D1A is required for the production of memory B cells in the development of long-term immunological memory [60].

On the other hand, we found *interleukin 8* (*il8*), *interleukin 1b* (*il1b*) and *interleukin 1 receptor type 2* (*il1r2*) to be up-regulated in both the ‘control challenged’ and ‘vaccinated challenged’ spleens, but the magnitude of increase was much smaller in the latter (**Table 4**). Interleukins 1 and 8 are known to be pro-inflammatory cytokines and this suggested that the level inflammation is possibly lower in the vaccinated spleens [61].

In addition to the elucidation of the functional genomic aspect of the seabass immune response, we have also demonstrated the possibility to semi-quantitatively measure the amount of pathogen directly from the holding water using a nested PCR method. Diseased fish are known to shed the pathogen into the tank water and the amount of the bacteria released into the water can be measured through bacteriological culture or antibody-based methods [62], [63]. The direct detection of pathogen from environmental samples using PCR based method has also been described by several other authors (see review: [64]). In addition to the detection of pathogen, this method has been used in the surveillance and detection of exotic Asian carp species in the Great Lakes of North America [65]. We found that while *S. iniae* could be detected to increase in the holding water of both challenged groups, there was less *S. iniae* in the vaccinated group (**Fig. 3**). It is likely that the increase in *S. iniae* in the ‘vaccinated challenged’ group is mostly due to multiplication of the *S. iniae* present in the water, while that of the ‘control challenged’ group mostly came from multiplication of *S. iniae* within the control seabass which was subsequently shed into the water. Hence, this result shows that vaccination not only led to a successful immune defense against the pathogen challenge, but also minimized the horizontal transmission of the pathogen through reduced bacterial shedding.

In conclusion, we have provided a transcriptomic overview of how the Asian seabass spleen and head kidney responded to vaccination and pathogen challenge. Our time-based analysis of the transcriptomes of the spleen and head kidneys after vaccination revealed that the two organs had very different responses that are suggestive of their different roles in establishing a vaccine-induce disease resistance. In addition, we partially uncovered the molecular mechanisms involved in the adaptive immune responses towards the pathogen challenge, particularly the role of NF-κB signaling. Based on the results, we speculated and provided a working hypothesis whereby the teleost spleen is mainly involved in the cellular response while the head kidney is mainly involved in the humoral response. It would be also useful to extend this study in future in elucidating the responses of the two organs towards viral and parasitic pathogens.

## Supporting Information

Table S1 List of primers used in qRT-PCR.
(DOCX)

Table S2 List of 938 transcripts that were differentially expressed in the spleen in D1 vaccinated group compared to D1 control group (p-value FDR<0.05; fold-change ≥1.5 or ≤−1.5).
(XLSX)

Table S3 List of genes up-regulated in the spleen in D1 vaccinated vs. D1 control groups and grouped according to the KEGG pathways (p-value FDR<0.05; fold-change > = 1.5).
(XLSX)

Table S4 List of 976 transcripts that were differentially expressed in the spleen in the vaccinated challenged group compared to the vaccinated mock group at 25–29 hpc (p-value FDR<0.05; fold-change ≥1.5 or ≤−1.5).
(XLSX)

Table S5 List of 12,157 transcripts that were differentially expressed in the spleen in the control challenged group compared to control mock challenged group at 25–29 hpc (p-value FDR<0.05; fold-change ≥1.5 or ≤−1.5).
(XLSX)

Table S6 Pathways with member genes that were up-regulated upon pathogen challenge in the control (un-vaccinated) spleens, but not in the vaccinated spleens, at 25–29 hpc when compared against their respective controls.
(XLSX)

Table S7 List of 577 transcripts that were differentially expressed between vaccinated (challenged and mock challenged) and control (challenged and mock challenged) head kidneys at 25–29 hpc (p-value FDR<0.05; fold-change ≥1.5 or ≤−1.5).
(XLSX)
