# Supplementary material for: Differential Transcriptomic Response in the Spleen and Head Kidney Following Vaccination and Infection of Asian Seabass with Streptococcus iniae
Source: PLoS One. 2014 Jul 3;9(7):e99128. doi: 10.1371/journal.pone.0099128 (PMC4081116; doi:10.1371/journal.pone.0099128)
Supplement: Table S1 — List of primers used in qRT-PCR. (DOCX) [file pone.0099128.s001.docx]

**Supplementary Table S1.** List of primers used in qRT-PCR.

| **S/N** | **KO** | **KO Title** | **Forward** | **Reverse** |
| --- | --- | --- | --- | --- |
| 1 | K02161 | *bcl2; B-cell leukemia/lymphoma 2* | GGTGAACTGGGGGCGTATC | GATGTCGTCCACCTGCCC |
| 2 | K05856 | *lck; lymphocyte cell-specific protein tyrosine kinase* | CTGGAGCGGGGATACAGAAT | TGTCCTCTGGGTTTTCCCTC |
| 3 | K07360 | *zap70; zeta-chain (TCR) associated protein kinase* | CTGGGAACTTTGGCTGTGTC | GCCTCCCTCATCATCTCCTC |
| 4 | K10785 | *trvb; T-cell receptor beta chain variable region* | CAGACCATGTCCGTGTGTTC | TTTAGCAGGGACCCTCAACC |
| 5 | K04179 | *ccr4; C-C chemokine receptor type 4* | GTCTGTTCGGCAACATCCTG | GTGGTAGTAGGCCCAGAAGG |
| 6 | K04182 | *ccr7; C-C chemokine receptor type 7* | AGCGTGGATCGTTATTTCGC | GCTGATGACACCTTGCTGAG |
| 7 | K04190 | *cxcr5; C-X-C chemokine receptor type 5* | AGAGAGCCTGGGTTTCTCAC | TGTGACCCTGAGCTCTGATG |
| 8 | K07990 | *sh2d1a; SH2 domain containing 1a* | AGTCGAGCACAAACACACAC | GTCCCTGAGTAGCATGTGGT |
| 9 | K05398 | *tlr1; toll-like receptor 1* | GGAGAGCATGGCAATCACTG | ATTCAAGGGTTTTGGCACCC |
| 10 | K05496 | *bmp3/3b; bone morphogenetic protein 3/3b* | AAAGCACCCTGCAAAGGAAG | TGAAACTGCGGACAGTGTTG |
| 11 | NIL | *gas7l; growth arrest-specific 7-like* | CGTTCAGAGGCGACAACTTC | ACGAGCCTTCTCTACACCTG |
| 12 | K03173 | *traf2; Tnf receptor-associated factor 2* | TTTACCAGGAATCCCCCTCAG | AGCAATGTACACAGAAGCGG |
| 13 | K08617 | *adamts1; ADAM metallopeptidase with thrombospondin type 1 motif, 1* | AACAATGCTGCTTCACCCAG | ACTCCCTCAAGGTCCATTCG |
| 14 | K05148 | *tnfrsf11b; tumor necrosis factor receptor superfamily member 11b* | ATGTGACCCTGTGTGAGGAG | TCTTACGATCCACCCTACGC |
| 15 | K10030 | *il8; interleukin 8* | GCCACCCTGAAAAAGACAGG | CTCAGCGTCTTCTGTTGGAC |
| 16 | K05443 | *il10; interleukin 10* | ATCTGCACTCTTGAGGACCC | ACAGACAGGAGAAGCAGGAC |
| 17 | K03996 | *c7; complement component 7* | CAACGCCTTGATAGGACACG | GGGGCAGAGTTTTGGAACTG |
| 18 | K04387 | *il1r2; interleukin 1 receptor type 2* | GGAGGAGGGTATCGAAGGTG | CTGTGCCACAACTTCCTGTC |
| 19 | K04519 | *il1b; interleukin 1b* | GTCATGCCACAAGAGTGGTG | CCGTACTGAAATGCGACAGG |
| 20 | K04358 | *fgf; fibroblast growth factor* | TCGAGACAATCCCCAGATCG | TGTTCAGACACACTCTCCCC |
| 21 | K05625 | *tgm2; transglutaminase 2* | TCTGGGAGTGAAGTACGACG | CTGTGATCTTCAGCCGTTGG |
| 22 | - | *rpl8; ribosomal protein L8* | AACTGCTGGCCACGTGTCCG | ACCTTGCGTCCAGCAGGTGC |
| 23 | - | *ubq; ubiquitin* | GCACACTGTCTGACTACAACATCC | CTTGTCGCAGTTGTATTTCTGG |
| 24 | - | *ef1a; elongation factor 1a* | CGCCACTGTTGCCTTTGTCCC | GGCATCCAGAGCTTCCAGCAGTG |
